# The push and pull factors: Adverse childhood experiences and their association with homelessness

**DOI:** 10.4102/hsag.v30i0.2847

**Published:** 2025-06-26

**Authors:** Noxolo C.M. Zwane

**Affiliations:** 1 Department of Social Work, College of Human Sciences, University of South Africa, Pretoria, South Africa

**Keywords:** homeless children, adverse childhood experiences, homeless, push, pull, substance use

## Abstract

**Background:**

A widespread global risk for children is homelessness, often caused by adverse childhood experiences (ACEs) and leading to dire living conditions. Children may be compelled to live and work on the streets because of economic, social and psychological factors. Knowledge of the patterns of ACEs and their association with homelessness remains unknown in South Africa.

**Aim:**

The study explores the patterns of ACEs contributing to childhood homelessness in South Africa to identify immediate causes and underlying factors that sustain the issue.

**Method:**

A qualitative document analysis was used as the primary method to review and interpret relevant academic publications. Thematic analysis was employed to analyse the data, providing a structured approach to organising, categorising and examining the material, enabling the identification of key patterns and themes that guided the study’s interpretation.

**Results:**

The study identified push and pull factors such as family dysfunction, substance use, street culture, peer influence and poverty that shape child displacement and contribute to pathways into homelessness.

**Conclusion:**

It was evident that family challenges, abuse and poverty push children from unsafe homes, while peer influence and street culture pull them into prolonged street life. Addressing the push-pull factors associated with ACEs requires a multifaceted approach that addresses the reasons why children leave their homes.

**Contribution:**

This article explores ACEs leading to child homelessness and offers insights for families, communities and policymakers to reduce the issue in South Africa.

## Introduction

### Background

According to the United Nations Educational, Scientific and Cultural Organization (UNESCO 2019) estimates, approximately 150 million children live and work on the streets across the globe. This figure highlights a global crisis, transcending borders and cultures, of the widespread nature of homeless children and underscores the urgent need for comprehensive, evidence-based interventions to address the complex challenges faced by street-connected children worldwide. Estimating the exact number of street children in South Africa is challenging because of their transient nature and the lack of comprehensive data (UNESCO 2019). Estimates indicate that there are over 150 000 street children in Ethiopia, around 30 000 in Accra, Ghana and between 250 000 and 300 000 homeless children in Kenya. In Nigeria, there are about 2 million homeless children, which is a concern for the various countries. This highlights the urgent need for targeted interventions and support to address the plight of these vulnerable young individuals based on evidence-based research (Skhosana, Schenck & Botha 2014).

Homeless individuals represent one of the most marginalised and vulnerable groups within society, often experiencing profound social exclusion, economic hardship and limited access to essential services (Kgadima & Mahlangu 2022). This phenomenon has been examined from multiple perspectives, leading to various interpretations (Gunhidzirai & Tanga 2020). Adverse childhood experiences (ACEs) are deeply impactful events or conditions during a child’s formative years, typically between birth and age 17 (Skhosana et al. 2014). These experiences encompass various forms of abuse, neglect and household dysfunction, such as physical, emotional or sexual abuse, exposure to domestic violence, parental substance abuse, mental illness and the incarceration of a household member (Skhosana et al. 2014).

Additionally, pull factors are conditions or attractions that draw people toward a new location or situation. These factors motivate individuals or groups to move or migrate because they promise better opportunities, improved living conditions, or other benefits (ILO, 2017).

This article aims to understand homelessness and its association with ACEs. Patterns of ACEs and their association with homelessness have not been thoroughly examined in South Africa.

Adverse childhood experiences refer to various traumatic events that occur during childhood, which can significantly impact an individual’s emotional, psychological and physical well-being (Crouch et al. 2017a). These experiences include abuse, neglect, household dysfunction and other forms of trauma (Crouch et al. 2017a). Understanding the patterns of ACEs is crucial, as they often serve as push factors that contribute to the risk of homelessness. Conversely, certain pull factors can mitigate or exacerbate this risk, influencing an individual’s path towards stability or instability (Crouch et al. 2017a). The drive to develop a comprehensive and accurate understanding of homeless children has led research to focus on the push and pull factors that bring children to the streets (Skhosana et al. 2014) because of ACEs. The need to conduct the study is confirmed by Nasir and Khalid (2015), who indicate that researchers and non-governmental organizations (NGO) struggle to maintain official statistics on the number of homeless children, as tracking their records is challenging because of the lack of identity documents or birth certificates, rendering them officially non-existent. In addition, there is a pressing need for a more in-depth exploration of the various childhood adversities measured within the ACE framework (Crouch et al. 2017b). Research conducted in the United States (US) in hospital settings highlights the value of incorporating ACE screening into public health strategies, emphasising the need for trauma-informed care in healthcare settings.

Adverse childhood experiences have been shown to significantly disrupt a child’s sense of security, with long-lasting effects on cognitive, emotional and social development, often limiting educational and socio-economic opportunities (Dube 2018). These outcomes can perpetuate intergenerational cycles of poverty and disadvantage. The present study explores how ACEs influence push and pull factors, contributing to pathways into homelessness. Push factors, such as abuse, neglect and family instability, force children out of their homes. At the same time, pull factors, such as the allure of perceived independence substance abuse, peer pressure or escape from trauma, draw them towards homelessness. The significance of this study lies in its potential to illuminate the underlying causes of homelessness among those with ACEs, emphasising the urgent need for targeted interventions. (Nazareth et al. 2022). For this study, the terminology homeless children will be utilised.

Policymakers and practitioners recognise that services and policies for homeless children are often fragmented and inadequate, making it challenging to develop effective interventions and build support for necessary reforms (Skhosana et al. 2014). The White Paper for Social Welfare (Department of Social Development 2021) highlights the growing challenges faced by families affected by domestic violence, substance abuse, mental health issues and poverty – factors that contribute to homelessness. It identifies youth and families as particularly vulnerable and calls for a multi-sectoral approach focusing on prevention and early intervention. The document also underscores the extensive literature on urban poverty and its impact on child homelessness in South Africa.

This unique angle of investigation aims to shed light on aspects that have not been thoroughly examined in previous research, thereby offering new insights into the challenges and dynamics experienced by this vulnerable population so that policies and interventions can be aligned with their current needs (Crouch et al. 2017a). Adverse childhood experiences are well documented as significant predictors of various adverse life outcomes, including homelessness. While the relationship between ACEs and homelessness is complex, the specific patterns of how push and pull factors contribute to this vulnerability remain underexplored. Understanding the interplay of these factors is crucial for developing targeted interventions to mitigate the long-term effects of ACEs and prevent homelessness among vulnerable populations (Crouch et al. 2017a).

### Aim

The study addresses the following research question: *What are the push and pull factors associated with ACEs that contribute to patterns of homelessness?*

## Methods

### Study design

A qualitative document analysis approach was adopted to gain a comprehensive understanding of the pull and push factors that are contributing to homelessness among children.

Document analysis, often overlooked in qualitative research (Kayesa & Shung-King 2021), offers valuable insights by systematically examining various documents (Sankofa 2022). This method helps address ethical concerns in other qualitative approaches (Morgan 2022), making it a valuable tool for sensitive or complex topics. The approach encompasses a rigorous and detailed review of printed and electronic documents, including personal and non-personal materials such as online articles and journals (Kayesa & Shung-King 2021). Deductive content analysis is applied when the analytical framework is established based on prior knowledge, and the study aims to test a specific theory (Elo & Kyngäs 2008).

### Document analysis process

The document analysis followed the four-step READ approach outlined by Dalglish, Khalid and McMahon (2020):

**Ready your materials:** To ensure a systematic approach to data collection, relevant documents were selected from reputable academic databases, including PsychINFO, SocINDEX, ProQuest Central, EBSCOhost, Google Scholar, ResearchGate, Academic OneFile, Sabinet, Scopus and PubMed. The search focused on scholarly articles, reports and other academic materials addressing key themes related to the study. A total of 100 relevant studies were identified by reviewing bibliographies of key papers. The search was limited to English-language documents published between 2014 and 2024. Documents meeting the inclusion criteria were thoroughly reviewed to extract data, identify recurring themes and assess their relevance to the research questions (Elo et al. 2014).

**Extract data:** The data extraction process used tools such as Microsoft Excel and Word to gather and systematically organise information relevant to the research question. This structured approach ensured that only data aligned with the study’s objectives were included. The organised data enabled the identification and interpretation of key patterns and themes that addressed the core aims of the research.

**Analyse data:** The data were analysed through skimming, reading and thematic analysis, organising information into categories aligned with the research questions. As noted by Creswell and Poth (2018), this iterative process allowed for the continuous refinement of findings. During this step, thematic analysis was used, where the information was organised into categories related to the central questions of the research. The identified themes were classified into two broad categories: *Factors pushing children to the streets* and *factors pulling children to the streets.*

**Evaluate findings:** Emerging themes were identified through focused re-reading and a detailed review of the data. Documents were read to extract specific data relating to the research question. A comprehensive literature search was conducted, reviewing bibliographies of key papers and identifying 100 relevant studies. After screening, 50 papers were deemed appropriate, with 30 offering future research recommendations and 24 highlighting strengths and limitations.

Trustworthiness was ensured through credibility, as outlined by Yilmaz (2013:321). Peer debriefing, used to gain insights and enhance data quality, was employed through ongoing communication with the research mentor throughout the study to verify the accuracy of the analysed documents.

#### Inclusion criteria

- Publications not older than 10 years.
- Publications that were in English.
- Publications focusing on ACE, homelessness, children who are homeless, substance use and social work related.

#### Exclusion criteria

- Publications older than 10 years.
- Publications that were not in English.

## Theoretical framework

**FIGURE 1:**
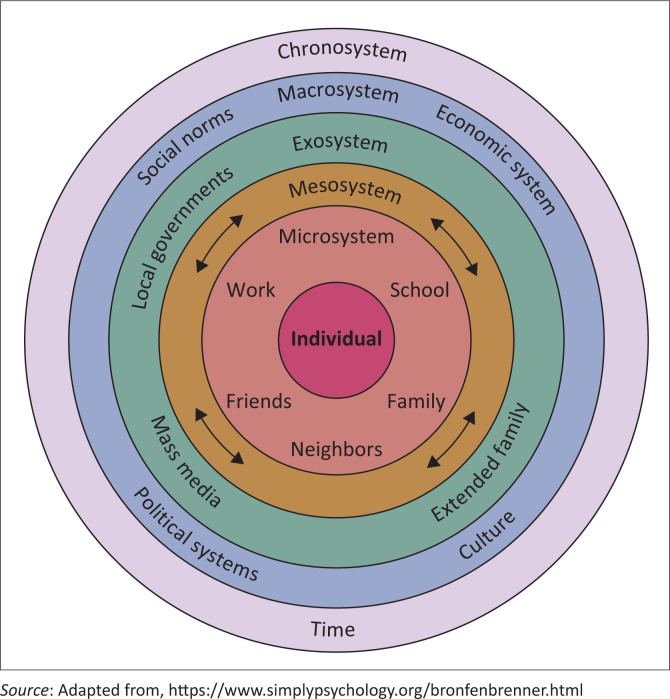
Illustration of ecological systems perspective.

The conceptual framework underpinning this study is the ecological systems perspective (Figure 1) developed by Urie Bronfenbrenner in the 1970s. Bronfenbrenner (1986) introduced the ecological systems perspective to explain how social environments influence development (Bronfenbrenner & Morris 2019).

The microsystem involves a street child’s immediate environment, such as family, peers and street networks, which exert positive and negative influences (Bronfenbrenner & Morris 2019). The mesosystem reflects interactions between these environments, affecting the child’s well-being. The exosystem includes external forces such as local policies and community resources indirectly influencing their lives. The microsystem encompasses broader societal and cultural norms. The chronosystem considers how life transitions, socio-historical events and ACEs shape the long-term trajectory of homelessness (Bronfenbrenner & Morris 2019; Guy-Evans 2024). The ecological systems perspective offers a valuable framework for understanding the complex challenges that drive children into homelessness. It highlights how ACEs such as abuse, neglect and household challenges function as key push factors rooted in the microsystem, including family and immediate social environments. When these systems fail, children may view the streets as a more stable alternative (Skhosana et al. 2014). This study analysed 50 articles, from which two central themes emerged through thematic analysis: *Various push factors* and *pull factors associated with homelessness.*

## Literature review based on document analysis

### Factors pushing children to the streets

Push factors are conditions or circumstances that compel children to leave their homes and communities, often against their will (Lund, McDaid & Saraceno 2021). These factors can include various forms of abuse, neglect, severe poverty and challenging family circumstances, which create an environment so hostile or uninhabitable that remaining at home becomes increasingly unbearable (Ford et al. 2021). From the documents that were analysed by the researcher, the push factors include the following themes:

#### Challenging family structures

Homelessness among children stems from various interconnected factors, primarily dysfunctional family dynamics and adverse socio-economic conditions. Key contributors include family breakdown because of divorce, death or incarceration, leading to instability and pushing children to the streets (Sultana et al. 2024). Poor parenting, characterised by a lack of warmth, supervision or authoritarian styles, creates unsupportive environments that heighten the risk of ACEs (Poulsen Georgi & Niclasen 2025; Radcliff et al. 2019). Additional risk factors include young or unprepared parents and non-traditional family structures. Push factors such as family conflict and a lack of support often intersect with pull factors such as street income opportunities (Dube 2018). Addressing this issue requires a comprehensive, multi-dimensional approach.

#### Abuse and neglect

When faced with abuse or neglect, children may feel an overwhelming sense of desperation that compels them to run away from home in search of safety and refuge (Gunhidzirai & Tanga 2020). This response is often rooted in ACEs, which profoundly impact a child’s mental and emotional development. The cycle of trauma and adversity, beginning within the home, thus extends into their life on the streets, underscoring the urgent need for interventions that address both the immediate and long-term consequences of ACEs, which this study aims to address (Sultana et al. 2024). Abuse within the home is a key factor contributing to the high prevalence of street children in South Africa. Research analysed indicates that a significant proportion of children leave their homes because of experiencing physical, sexual or emotional abuse. Specifically, studies suggest that approximately 80% of street children have a history of such abuse (Stats SA 2018).

#### Poverty

Poverty within families is a profound and pervasive factor that drives many children to live and work on the streets (Nasir & Khalid 2015). Faced with insurmountable challenges, parents often find themselves unable to provide the necessities for their children, leading to situations where children feel compelled to leave the family home in search of a better life on the streets (Nasir & Khalid 2015). Poverty is intricately linked to unemployment, particularly in regions where seasonal work is a primary source of income. These pressures not only force children out of school but also push them towards a life on the streets, where they face the harsh realities of homelessness (Sultana et al. 2024). The cycle of poverty, marginalisation and vulnerability creates an environment where street life becomes both a symptom and a cause of further decline, making it imperative to address these issues at their root (Nasir & Khalid 2015). Poverty is a significant push factor contributing to homelessness, with its effects exacerbated by ACEs. Individuals living in poverty often face severe financial instability, leading to an inability to secure stable housing (Sultana et al. 2024). The combined impact of poverty and ACEs creates a precarious situation where the lack of financial resources, compounded by the emotional and psychological scars of past traumas, significantly increases the likelihood of becoming homeless (Sultana et al. 2024). By examining these factors, we can gain a more comprehensive perspective that encompasses all the elements influencing the matter.

### Factors pulling children to the streets

Pull factors refer to the conditions, opportunities or circumstances that actively entice children towards a life on the streets (Ford et al. 2021). These factors often create an allure that draws children away from their homes and communities, leading them to embrace street life (Ford et al. 2021).

#### Substance use

Substance use plays a critical role in influencing the lives of children who end up homeless, acting both as a push and a pull factor. The relationship between substance use and homelessness is bidirectional, whereby substance use can act as a catalyst for homelessness. Conversely, the experience of homelessness can further exacerbate substance use disorders (Phiri, Gloeck & Musekiwa 2023). The research analysed demonstrates a high prevalence of substance use disorders within homeless populations, with estimates indicating that approximately 75 000 individuals who inject drugs are homeless in South Africa, particularly in urban centres such as Cape Town, Durban and Pretoria (Phiri et al. 2023). The persistent nature of this problem has become a growing concern for a wide range of stakeholders, including families, community leaders, educators, social workers, healthcare professionals, academics and government officials (Cumber & Tsoka-Gwegweni 2016). In addition to these local entities, international development partners are increasingly focused on addressing the issue. The plight of homeless children is of significant concern, as they are often perceived to be at an elevated risk of succumbing to various social and health crises, including drug abuse (Cumber & Tsoka-Gwegweni 2016). Substance use among homeless youth is influenced by factors such as gender, age, homelessness duration and peer networks (Hills, Meyer-Weitz & Asante 2016).

#### Peer influence

Peers, including friends and siblings already experiencing homelessness, often influence children to join the street life, offering a sense of belonging that may be absent elsewhere. This dynamic increases the risk of exposure to harmful substances because of peer pressure and the desire for social acceptance (Gunhidzirai & Tanga 2020). The sense of community on the streets can make homelessness more tolerable, deterring children from seeking help or returning to stable environments. Peer influence also reinforces negative behaviours such as substance abuse and petty crime, making it harder for children to escape the cycle of homelessness (Gunhidzirai & Tanga 2020). Peer pressure can lead children to run away from home, with some reporting they were directly influenced by friends (Shitindi, Zhang & Nyello 2021). The absence of social support services further exacerbates this issue, pushing children towards illegal activities. In addition, the allure of street life, seen as more exciting than their home environments, can drive children towards substance use. The growing prevalence of drug abuse among homeless youth in South Africa is a significant concern, leading to long-term health issues, mental distress and increased social exclusion and criminal involvement (Sultana et al. 2024).

#### Street culture

Street culture plays a crucial role in shaping the experiences of children living on the streets, often as a pull factor that draws them into street life. Characterised by the norms, values and behaviours prevalent among street-involved youth, this culture offers a sense of belonging, financial independence and the appeal of urban life (Gunhidzirai & Tanga 2020). The desire for autonomy, the allure of freedom and the perceived glamour of city living can attract children to the streets, where they seek acceptance and companionship within peer groups. However, while these pull factors influence children’s decisions to engage in street life, they are often less significant than push factors such as poverty, family breakdown and abuse. The interplay between these push and pull factors highlights the complex dynamics that drive children towards life on the streets (Phiri et al. 2023).

## Discussion

Two key elements allow this study to stand out: the push and pull factors that play a relevant role in children living on and off the street with ACEs. In the review of the literature for this study, it is evident that homeless children are impoverished, vulnerable and often perceived as dysfunctional, markedly different from other children (Phiri et al. 2023). The *lack of adequate support* provided to homeless children encompasses a range of difficult circumstances they may face at home. These challenges often include parental or caregiver unemployment, alcoholism, domestic violence, abuse and exploitation, which push them to become homeless (Gunhidzirai & Tanga 2020). As a result, many children are compelled to seek out social connections and networks on the streets, where they believe they might find care and community absent in their home lives, and they remain on the street for prolonged periods (Gunhidzirai & Tanga 2020).

In addition, *poverty and other economic pressures* play a crucial role as significant factors driving children familiar with ACEs into homelessness. The harsh reality is that economic hardships, combined with the inability of families to meet even their most basic needs, often compel many children to leave their homes in search of survival on the streets (Dladla & Ogina 2018). This combination of socio-economic challenges and emotional deficiencies creates a grim picture of the reality faced by homeless children, illustrating their acute vulnerability and the intricate interplay of factors leading to their lives on the streets (Dladla & Ogina 2018). In addition to the physical risks, homeless children also face significant psycho-social challenges. Understanding how their environment influences their well-being is vital, emphasising the importance of addressing the physical and psycho-social factors contributing to their vulnerability. Recognising and addressing these issues are essential for developing effective interventions that can improve the lives of homeless children, help break the cycle of poverty and disadvantage they endure and assist with appropriate ACE-related interventions (Dladla & Ogina 2018).

The research analyses confirm that when parents or caregivers struggle with *dependency,* they can unintentionally create a home environment that is unsafe and unstable. This instability often results in what is known as the ‘push effect’, where children are driven away from their homes. The problem of drug and substance abuse is multifaceted and continuously evolving, reflecting its complex nature. For homeless children, drugs offer a temporary escape from their harsh realities. They provide a fleeting sense of relief from their daily struggles, bolster the courage to engage in criminal activities, facilitate sleep and supply the necessary strength to undertake various economic activities crucial for survival. This widespread issue highlights the profound impact that drug abuse has on the lives of homeless children, as it becomes an integral part of their daily routines and survival strategies (Cumber & Tsoka-Gwegweni 2016).

The ecological systems perspective offers a comprehensive framework to understand the push and pull factors influencing homelessness among individuals with a history of ACEs. The microsystem plays a crucial role, with early experiences of abuse, neglect and family dysfunction creating an unstable environment that disrupts child development. These push factors isolate individuals, making them vulnerable to homelessness because of a lack of support systems. Pull factors, such as perceived benefits of street life, draw individuals towards unstable environments. At the exosystem and macrosystem levels, poverty, inadequate housing and systemic inequality contribute to homelessness. In addition, the chronosystem highlights the long-term effects of ACEs, with cumulative disadvantages reinforcing pull factors as individuals age. Understanding these factors holistically is vital for shaping effective interventions for homeless children and guiding policymaking.

## Recommendations

The implications of this study encompass the following recommendations for social work practice:

### Understanding the push-pull effect

Understanding the push-pull dynamics is key to developing effective interventions and policies. Recognising the factors driving children away from home and attracting them to the streets is essential for creating targeted solutions. Increasing awareness of ACEs through community workshops, school programmes and training for educators and caregivers can be effectively implemented by social workers.

### Comprehensive support systems

An extensive support system for families is crucial in addressing poverty, preventing abuse and strengthening relationships, thus helping mitigate factors contributing to child homelessness. Expanding access to mental health services and counselling for children and families affected by ACEs promotes resilience and well-being. In addition, tailored early intervention and prevention programmes should address specific family needs, ensuring practical support based on recent research.

### Community awareness and inclusive programmes

Enhancing community awareness and implementing inclusive programmes are key strategies for addressing family breakdown and homelessness. Proactive community engagement and policy advocacy focused on poverty alleviation and equitable access to healthcare and education create a supportive environment for at-risk families. Educating communities fosters empathy, while social workers play a vital role in organising initiatives that promote inclusivity, strengthen social connections and empower families to overcome adversity.

## Conclusion

Key push factors such as family issues, substance abuse, domestic violence and poverty create unsafe home environments that drive children to the streets. Pull factors such as peer networks and perceived safety in street life prolong homelessness. These children face severe physical, emotional and psychological risks. The study’s findings will guide early intervention and prevention strategies tailored to these factors for a more effective response to childhood homelessness.

### Recommendations for future research

Addressing homeless children’s plight requires understanding the factors behind their displacement. Effective intervention must meet immediate needs while tackling the root causes of homelessness and vulnerability, underscoring the need for further research to inform targeted, long-term solutions.

The goal is to implement targeted interventions that address contributing factors and yield positive outcomes, ultimately creating safer, more supportive environments for homeless children. In summary, addressing the push-pull factors of ACEs requires collaboration among families, communities, schools and policymakers to foster a nurturing environment for all children.
